# Free-Standing, Water-Resistant, and Conductivity-Enhanced PEDOT:PSS Films from In Situ Polymerization of 3-Hydroxymethyl-3-Methyl-Oxetane

**DOI:** 10.3390/polym16162292

**Published:** 2024-08-14

**Authors:** Sara M. Jorge, Luís F. Santos, Maria João Ferreira, Carolina Marto-Costa, Ana Paula Serro, Adelino M. Galvão, Jorge Morgado, Ana Charas

**Affiliations:** 1 Instituto de Telecomunicações, Instituto Superior Técnico, Av. Rovisco Pais, 1049-001 Lisboa, Portugal; 2 Centro de Química Estrutural, Departamento de Engenharia Química, Institute of Molecular Sciences, Instituto Superior Técnico, Universidade de Lisboa, 1049-001 Lisboa, Portugal; 3 Egas Moniz Center for Interdisciplinary Research (CiiEM), Egas Moniz School of Health & Science, Campus Universitário, Quinta da Granja, Monte da Caparica, 2829-511 Almada, Portugal; 4 Department of Bioengineering, Instituto Superior Técnico, Universidade de Lisboa, 1049-001 Lisboa, Portugal

**Keywords:** PEDOT:PSS, free-standing films, oxetane, conducting polymers, electrical conductivity

## Abstract

Free-standing films based on conducting polymers, such as poly(3,4-ethylenedioxythiophene): poly(styrene sulfonate) (PEDOT:PSS), offer many benefits over traditional metal electrodes for applications in flexible electronics. However, to ensure structural integrity when contacting aqueous environments and high levels of electrical conductivity, solution-processed polymers require additives that act as crosslinking agents and conductivity enhancers. In this work, a new approach is presented to fabricate water-resistant free-standing films of PEDOT:PSS and simultaneously increase their conductivity, using an oxetane compound as an additive. It is shown that at moderate temperatures, oxetane polymerizes within the PEDOT:PSS acidic medium, forming hydroxymethyl-substituted polyether compounds that form a network upon crosslinking with PSS. The polymer composite films show self-sustainability, structural stability in aqueous environments, and enhanced conductivity. Finally, the potential of the free-standing films as health-monitoring electrodes, specifically for human electrocardiography, is explored.

## 1. Introduction

Conjugated polymers (CPs) represent a very interesting class of materials due to their unique combination of electrical properties, which can be similar to those of inorganic semiconductors and metals, with properties of plastics, such as mechanical flexibility, lightweight structure, and low-cost processing, including deposition from a solution (such as inks). Among the variety of electroactive polymeric systems synthesized, PEDOT:PSS (poly(3,4-ethylenedioxythiophene):poly(styrene sulfonate) is one of the most studied, because it produces thin films with high optical transparency, excellent thermal stability, electrochemical activity, and tunable electrical conductivity (from ca. 10^−3^ Scm^−1^ to over 4300 Scm^−1^) upon the implementation of adequate doping procedures [1,2]. For comparison, the conductivity of copper, 5.96 × 10^5^ Scm^−1^ (at 20 °C), is approximately one order of magnitude higher. Furthermore, PEDOT:PSS is commercially available as aqueous dispersions with different formulations (e.g., various PEDOT:PSS weight ratios), offering easy processing by solution casting techniques, such as spin-coating. A remarkable example of PEDOT:PSS applications as thin films concerns its capability to replace the widely used transparent electrodes made of indium–tin–oxide (ITO), whose replacement is urgent due to the declining availability of indium [3,4]. Most recently, PEDOT:PSS has attracted significant interest as an electroactive component for an emerging generation of devices in the bioelectronic field, such as implantable devices, electrochemical transistors functioning as biosensors, and cutaneous electrodes, among others [5,6].

In the pursuit of the high conductivity required for thin-film electrodes, numerous additives of different classes have been explored, particularly protic acids (e.g., sulfuric acid [7], methane sulfonic acid [8], formic acid [9]), alcohols (e.g., ethylene glycol [10], sorbitol [11]) and other polar solvents (e.g., DMSO [12], DMF [13]), and ionic liquids [14]. In applications involving contact with aqueous media, the electrode water resistance is crucial. Unfortunately, the structural integrity of pristine PEDOT:PSS films tends to be insufficient in such conditions. PEDOT:PSS films often experience cracking, redispersion, or delamination due to the hydrophilic nature of the PSS polyelectrolyte [15,16]. To overcome these drawbacks, a common strategy involves incorporating crosslinking agents into PEDOT:PSS aqueous dispersions, for which a variety of substances, including glycerol [17], poly(ethylene oxide) (PEO) [18], divinyl sulfone (DVS) [19], ionic azides [20], and siloxanes (e.g., glycidoxypropyltrimethoxysilane (GOPS) [21]), have been proposed. However, despite their success as crosslinkers, most treatments lead to only a modest increase in the film conductivity or even result in its reduction. When both structural integrity and enhanced electrical conductivity are required, strong acids such as H_2_SO_4_ and H_3_PO_4_, applied in solution or vapors in PEDOT:PSS films’ post-treatments, have proved successful [22,23]. This approach enabled reaching conductivity levels surpassing 4000 Scm^−1^ in PEDOT:PSS thin films treated with H_2_SO_4_ [23]. Nevertheless, despite their efficacy, treatments with strong acids entail shortcomings, particularly in applications where PEDOT:PSS interfaces directly with biological media, since acids can modify the surface charge of PEDOT:PSS films, potentially impacting biological events. In addition to enabling highly conducting thin films (whose thicknesses are tens of nanometers) casted on substrates, PEDOT:PSS is very promising for free-standing films for applications within diverse areas, such as wearable electronics [24], tissue engineering [25], and MEMs (Microelectromechanical Systems) [26].

Although PEDOT exhibits minimal mechanical stretchability in its pristine form, owing to its rigid conjugated backbones and their relatively robust interchain interactions, its organic nature facilitates the development of flexible, conformable, and stretchable electronic components. The improvement of PEDOT:PSS’s mechanical robustness enabling free-standing films has been shown through the incorporation of small molecules or polymers acting as “plasticizers” [27]. In particular, a few specific small molecules can act as plasticizers by reducing the interactions between polymer chains and increasing the free volume, which leads to a decrease in the elastic modulus [28]. In this category, several compounds were explored, such as ethylene glycol [29], D-sorbitol [30], and ionic liquids [31,32], which led to conductivity enhancements as well. For example, He et al. fabricated PEDOT:PSS free-standing films exhibiting a conductivity of around 1000 Scm^−1^ by mixing PEDOT:PSS PH1000 with D-sorbitol and then drop-casting the mixture on glass substrates [30]. After drying at 80 °C, free-standing films with tens of micrometers of thickness were obtained. Teo et al. fabricated PEDOT:PSS free-standing films with thicknesses above 300 μm by drop-casting a mixture of PEDOT:PSS PH1000 and an ionic liquid, 1-ethyl-3-methylimidazolium tetracyanoborate (EMIM TCB), in polytetrafluoroethylene (PTFE) molds supported on silicon wafers [31]. The samples were then left in ambient air for a few days for solvent evaporation and then dried at 60 °C before they could be peeled off from the substrates. One disadvantage associated with the use of small molecules as additives is their easy leaching into the environment, which may result not only in the degradation of the material properties but also in creating health risks. Concerning the blending of polymers as plasticizers with PEDOT:PSS, the additive polymer has to be soluble and chemically stable in the PEDOT:PSS acidic solution. Therefore, only a few polymers have been explored, namely, poly(vinyl alcohol) (PVA) [33], poly(ethylene glycol) (PEG) [33], and waterborne polyurethane (WPU) [34]. Blending insulating polymers as plasticizers of PEDOT:PSS induces a decline in conductivity as the proportion of the insulating polymer increases, driven by a phase separation that restricts the connectivity between the conductive PEDOT domains [34]. By employing the polymer blending approach, Li et al. explored three different soft polymers (PEG, PEO, and PVA) in mixtures with PEDOT:PSS that were drop-casted on glass substrates and then dried at 70 °C [33]. After cooling, the films could be peeled off from the substrates, and those made of PEDOT:PSS:PEG exhibited the highest conductivity: 101 Scm^−1^ [33]. Impressively, in a different work, the mixing of PEG with PEDOT:PSS was found to result in highly conducting free-standing films after post-treatment with water, according to Sun et al. [35]. The authors drop-casted mixtures with PEG with different molecular weights onto glass substrates, dried them for about 2 h, and soaked the films in water for consecutive periods of one hour. After drying the soaked films at 120 °C, they obtained free-standing films approximately 8 μm thick reaching conductivities as high as 1415.7 Scm^−1^. To attain excellent conductivities, Li et al. produced a paste of PEDOT:PSS plates by mixing PEDOT:PSS PH1000 with dilute H_2_SO_4_ by high-speed stirring [36,37]. The paste was then casted onto filter paper, from which the liquid filtrate was separated. The resultant film was immersed in acetone and thereafter immersed in a concentrated H_2_SO_4_ solution for a week to achieve a conductivity of 2500 Scm^−1^ in ca. 3 μm thick free-standing films. Other authors used a combination of additives as a strategy to improve both the conductivity and the mechanical robustness of PEDOT:PSS films. For example, Jin et al. [38] used dilute H_2_SO_4_ to prepare a PEDOT:PSS paste, similarly to Li et al. [36], and then polyethylene glycol 2,5,8,11-tetramethyl 6-dodecyne-5,8-diol ether (PEG-TmDD) as an additive to improve the wetting properties of the free-standing film. However, the conductivities, about 336 Scm^−1^, measured in films with a thickness of around 6 μm were much lower than those obtained for the samples prepared employing only H_2_SO_4_.

Recently, we explored oxetanes, namely, 3-hydroxylmethyl-3-methyl-oxetane (HMO), 3-ethyl-3-oxetanemethanol, 3-oxetanol, 3-oxetanylmethanol, 3-(chloromethyl)-3-methyloxetane, and 3,3-dimethyloxetane, as additives to improve both the water resistance and conductivity of PEDOT:PSS thin films [39,40,41]. Oxetanes are a class of heterocycle organic compounds that have recently received huge interest as intermediates in organic synthesis, especially for drug development, for being, in general, nontoxic and biocompatible [42,43]. We found that when PEDOT:PSS:oxetane mixtures are applied through spin-coating onto glass substrates to form thin films, if the oxetanes contain hydroxyl groups in the substituents of the oxetane ring, a significant increase in the conductivity of the PEDOT-based films, by more than three orders of magnitude, is obtained [39]. In addition, the films´ transmittance in the visible range remains nearly unaffected or is even improved. Through ^1^H-NMR studies, we found that the oxetanes undergo polymerization during the film-drying step carried out at 120 °C, resulting in polyether chains [39,40]. Moreover, the spectra showed that hydroxyl groups within the newly formed polyether chains can undergo esterification with sulfonate groups from HPSS, thereby leading to covalent crosslinking with PSS. We attributed the enhancements in conductivity to the screening of the negative charge of PSS as a result of esterification reactions or interactions with hydroxyl groups from polyether chains. Consequently, conducting PEDOT chains segregate and form more ordered domains where charge transport is facilitated. Notably, the polymerization of the oxetanes and the crosslinking reactions take place exclusively during the film-drying stage, therefore ensuring that the properties of the formulated mixtures are not affected by any possible gelation or precipitation that could arise from the oxetanes’ polymerization [39].

**Figure 1:**
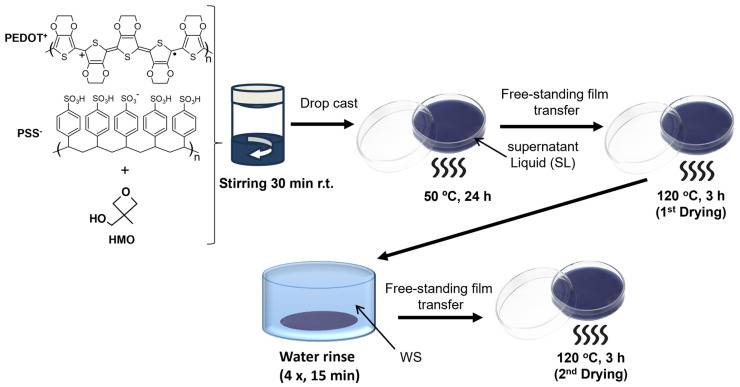
A sketch of the preparation of free-standing films from PEDOT:PSS:HMO mixtures.

Here, we explore a hydroxyl-substituted oxetane compound, HMO (Figure 1), to produce free-standing films of PEDOT:PSS with electrical and mechanical properties adequate for cutaneous electrodes. More specifically, HMO was used as an additive to PEDOT:PSS PH1000 commercial dispersions. In contrast with the pristine PEDOT:PSS sample, free-standing films could be obtained after subjecting the mixtures casted on Petri dishes to a temperature range from 50 to 120 °C. Also, the films do not fragment when immersed in water. To further clarify the reactivity of HMO within the PEDOT:PSS medium, we conducted NMR studies at various stages of the film-forming process, and the films’ properties were investigated before and after their water-rinsing treatment and upon drying at 120 °C (Figure 1) to investigate the potential effect of water rinsing. In addition, most conducting films were tested as dry, flexible electrocardiography (ECG) electrodes to assess their potential for wearable electronics applications.

## 2. Materials and Methods

### 2.1. Materials

PEDOT:PSS aqueous dispersion Clevios™ PH1000 with a solid content 1.0–1.3 wt.% and a PEDOT:PSS ratio = 1:2.5 was acquired from Ossila (Leiden, The Netherlands). 3-Methyl-3-oxetanemethanol (HMO) was acquired from Merck (Darmstadt, Germany) and used as received.

### 2.2. Preparation of PEDOT:PSS:HMO Free-Standing Films

Mixtures of the PEDOT:PSS aqueous dispersion with HMO at different concentrations (0.005; 0.015; 0.05 *v*/*v*) were prepared by adding different volumes of HMO to 4 mL of the PEDOT:PSS dispersion through a microsyringe. The mixtures were stirred vigorously at room temperature for 30 min and were then deposited on glass-covered Petri dishes. The samples were heated at 50 °C on a hotplate in air for 24 h. During this period, thick free-standing films formed within a light blueish supernatant liquid (SL in Figure 1). Afterward, the films were transferred to Petri dishes lined with PTFE foil. The films were dried at 120 °C for 3 h in air and then peeled off into a beaker containing approx. 100 mL of deionized (DI) water. The films underwent a water-rinsing process in which the DI water was renewed 4 times after periods of 15 min. The films were then again transferred to Petri dishes lined with PTFE foil and they were dried at 120 °C for 3 h in air. At least eight samples were prepared with the same HMO ratio. To prepare reference samples from pristine PEDOT:PSS, the pristine dispersion was deposited on glass coverslips and heated at 50 °C on a hotplate in air for 24 h. Due to their dispersibility in water, the subsequent treatments performed on the PEDOT:PSS:HMO samples were not applied.

### 2.3. Material Characterization

#### 2.3.1. ^1^H and ^13^C Nuclear Magnetic Resonance (NMR)

The samples consisted of the residues in the supernatant liquid (SL in Figure 1) and in the washing solutions resulting from the water-rinsing step (WS in Figure 1) after removing the solvent by rotary evaporation. The samples were then dissolved in D_2_O for the analysis. In general, three samples of each formulation were collected and treated jointly in order to obtain a sufficient amount for the analysis. A Bruker Avance III 400 NMR spectrometer (Ettlingen, Germany) operating at 400 MHz equipped with a 5 mm two-channel (^1^H and X) Broad-Band probe was used for the Nuclear Magnetic Resonance (NMR) experiments. The ^1^H NMR spectra were referenced internally using the residual protio-resonances of the solvent to tetramethylsilane (TMS) (δ = 0). ^13^C{^1^H} spectra were referenced externally using the carbon resonances of THF relative to TMS (δ = 0).

#### 2.3.2. Fourier-Transform Infrared Spectroscopy

The infrared spectra of the films were determined using a Thermo Electron Nicolet 5700 Attenuated Total Reflectance (ATR)-FTIR instrument (Thermo Electron, Waltham, MA, USA) and an Alpha FTIR spectrometer (Bruker, Ettlingen, Germany) for transmission measurements. For transmission, the films were cut into small pieces, mixed with fine KBr powder, and then crushed for pellet formation. All the spectra were acquired with 4 cm^−1^ resolution and 128 scans.

#### 2.3.3. Electrical Conductivity Measurements

The electrical conductivities of the films were determined by the four-contact method using a Keithley 2400 Source Meter unit (Solon, OH, USA) and a multimeter (Agilent 34401A 6½ Digital Multimeter (Santa Clara, CA, USA). The films were coated with Au (40 nm) thermally evaporated under high vacuum (<10^−4^ mbar) through a shadow mask defining four gold stripes separated by channels with L = 300 μm. The DC-reversal technique was used to enhance accuracy and cancel out the effects of contact potentials. The film thickness was measured using a digital caliper (Schut Geometrical Metrology).

#### 2.3.4. Differential Scanning Calorimetry (DSC)

Thermal analysis was performed using a Differential Scanning Calorimeter (DSC) (Netzsch DSC 200 F3 Maia, Selb, Germany). The samples were sealed in aluminum pans. A small hole was made in the cap to allow a nitrogen flow within the capsule. The first heating, from 20 °C to 125 °C, was performed to promote the evaporation of any residual water. The samples were then subjected to one heating–cooling cycle between 20 °C and 400 °C at a heating/cooling rate of 10 °C/min under a nitrogen purge.

#### 2.3.5. Atomic Force Microscopy (AFM)

Atomic Force Microscopy analysis of the films was carried out using a Nano-Observer microscope (Concept Scientific Instruments, Les Ulis, France) operating in intermittent contact (tapping mode). Silicon tips with a ca. 10 nm end radius (ACT App. Nano from Concept Scientific Instruments, Les Ulis, France) were used as probes. All images were acquired with 256 × 256-pixel resolution and processed with Gwyddion software (version 2.56).

#### 2.3.6. Mechanical Properties

The mechanical behavior of the films under tensile stress was evaluated using a TA.XT Express Texture Analyzer (Stable Micro Systems, Godalming, Surrey, UK) equipped with a 50 N load cell. Uniaxial tensile tests were conducted at room temperature (≈25 °C) with a crosshead displacement speed of 1 mm/min until failure using rectangular test specimens (5 mm in width and 25 mm in length). The gauge length was 15 mm, and the thickness was as specified in Table 1. Each test was performed at least in quadruplicate. Young’s modulus was calculated from the slope of the stress–strain curve. Ultimate tensile strength and elongation at break were determined as the maximum stress and strain values from the stress–strain curves, respectively.

#### 2.3.7. Electrocardiogram (EGC) Recording

The EGC assays were conducted using a commercially available Movesense Medical single-channel ECG sensor (Movesense Ltd., Vantaa, Finland). One healthy female subject whose informed consent was obtained collaborated in this study. The recordings were made by having the left and right thumbs touching the left and right electrodes, respectively, of the Movesense cage. To test the films as electrodes, two identical films covered the fingertips of the user and interfaced with the electrodes of the sensor. Each experiment lasted approx. 30 s in the sitting (rest) position. The ECG tracings were recorded by the Movesense showcase app via a smartphone and were converted into .csv files.

## 3. Results and Discussion

Figure 1 shows the stepwise process carried out to prepare free-standing films from mixtures of PEDOT:PSS and HMO in various ratios (HMO/PEDOT:PSSaq. = 0.005, 0.015, and 0.050 *v*/*v*). As indicated, free-standing films were obtained after heating the drop-casted mixtures at 50 °C for 1 day. Also, a supernatant liquid was produced. The films were then transferred to PTFE-lined Petri dishes and dried at 120 °C for 3 h. Afterward, they were peeled off and transferred to a beaker with water, where they were subjected to a water-rinsing process, and they were dried again at 120 °C (see experimental section for details). During the rinsing process, the films kept their original form, therefore showing high water resistance. Figure S1 in Supplementary Materials shows films obtained at different stages of their preparation and includes a photograph showing their compliance with a curved surface. Notably, analogous volumes of pristine PEDOT:PSS dispersions treated at 50 °C and at 120 °C for similar periods yielded powdered samples, failing to form free-standing films. Therefore, such samples were characterized while deposited over glass substrates and were not submitted to the water-rinsing step since they lack structural integrity and re-disperse in water.

### 3.1. Electrical Conductivity of PEDOT:PSS:HMO Free-Standing Films

Table 1 shows the electrical conductivities and thicknesses of free-standing films prepared from mixtures of PEDOT:PSS and HMO in various ratios, measured before and after water rinsing and upon drying at 120 °C in each condition. For comparison, the results found for the pristine PEDOT:PSS sample are also shown.

The conductivity of the free-standing films is significantly enhanced in comparison with that of pristine PEDOT:PSS samples. In addition, while non-rinsed films show a decreasing trend in conductivity with increasing HMO content, the rinsed ones exhibit maximum conductivity when prepared from the mixtures containing the highest HMO load. The maximum conductivity achieved, around 106 Scm^−1^, is at the same magnitude as that reported by Li et al. [33] for free-standing films prepared by drop-casting mixtures of PEDOT:PSS and PEG20K (101 Scm^−1^). However, the attained enhancements fall behind those found in our previous studies for spin-cast films, for which the conductivity was 189 Scm^−1^ for films prepared from mixtures with HMO/PEDOT:PSS = 0.050 and reached 202 Scm^−1^ for more concentrated mixtures. As observed, the water-rinsing step increases the conductivity of the films for all formulations, with a more substantial effect on films prepared from mixtures with higher contents of HMO. These films are the least conductive among the non-rinsed ones, excluding the pristine PEDOT samples. Several studies have documented enhanced conductivity in PEDOT:PSS films following treatment with water [44,45]. The justifications for such improvements lie mainly in morphological alterations within the conductive PEDOT assemblies, ascribed to the removal of PSS. In our study, we inspected the composition of the washing solution by NMR and did not find the presence of PSS. This is discussed in the following section. Besides providing structural stability to PEDOT:PSS and enabling self-sustainability, the addition of HMO also produces an increase in the films’ thicknesses. Films with very high thicknesses, around 200 μm, were obtained for mixtures with higher HMO content. Nevertheless, after water-rinsing (and upon drying at 120 °C), the films exhibit lower or comparable thickness values, suggesting the removal of some material. For this reason, the composition of the washing solution (WS) was analyzed by NMR.

### 3.2. Reactivity of HMO in PEDOT:PSS Medium

**Figure 2:**
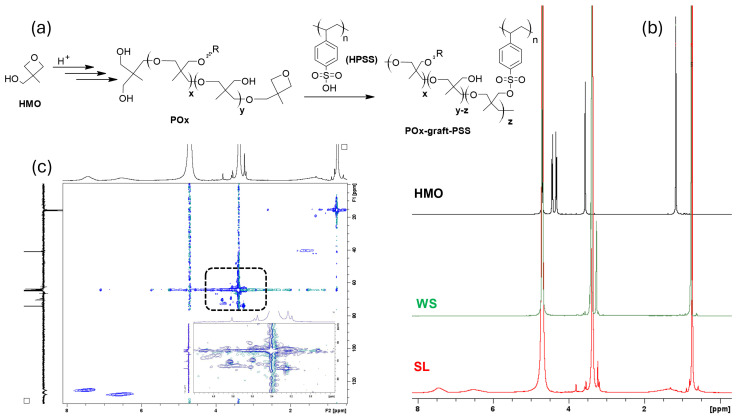
General structures of polyether chains formed from HMO in PEDOT:PSS acid medium (**a**); ^1^H NMR spectra of HMO, SL, and WS (**b**), and HSQC spectrum of SL in D_2_O (**c**). HPSS stands for polystyrene sulfonic acid.

In order to unravel the effect of HMO on the films’ properties, its reactivity within the PEDOT:PSS medium was investigated by NMR. Therefore, the following samples were collected for analysis: the supernatant liquid remaining after the annealing step (SL) and the washing solution resulting from the water-rinsing step (WS). For the analysis, the solutions were dried by rotary evaporation, and the solid residues were dissolved in D_2_O. Because the films were nearly insoluble, they were not included in these studies. In Figure 2, the ^1^H NMR spectra of the SL and WS samples and that of HMO are compared, and the Hydrogen–Carbon Correlation (HSQC) spectrum of the SL sample is also shown. Figures S2–S4 in Supplementary Materials show the ^13^C APT NMR spectra of the SL and WS and the HSQC spectrum determined for the WS. Because the spectra of samples differing in the HMO:PEDOT:PSS ratio in the mixtures diverge only in their signal-to-noise ratio and reflect mostly the change in the mixtures´ compositions, only the spectrum for one composition (HMO/PEDOT:PSSaq. = 0.05, *v*/*v*) is shown.

The ^1^H NMR spectrum of the SL is dominated by the characteristic peaks of polyether chains formed by the polymerization of HMO [39], together with the signals of PSS (three broad signals peaking at 7.46 and 6.52 ppm, attributed to the CH groups in aromatic rings, and at 1.47 ppm, attributed to the hydrogens in the aliphatic chains). By comparison with the spectrum of neat HMO, it can be concluded that HMO was completely consumed to yield the corresponding polyether compounds. The general formula of the polyether is determined considering the cationic ring-opening polymerization (CROP) that oxetanes undergo in an acidic medium [46] and the esterification reaction between the pendant hydroxyl groups in the polyether backbones and sulfonate groups in HPSS chains (Figure 2a). Hence, near the intense peaks at 3.37 ppm, which are attributed to the most abundant ether groups in the polyether chains (−CH_2_−O−CH_2_−), less intense signals within the range 3.81–3.19 ppm indicate the presence of hydroxymethyl-substituted blocks (−CH_2_OH), ether-terminated blocks (−CH_2_OR), and crosslinking nodes (−CH_2_OS−) with PSS. These assignments are in accordance with those determined in our previous studies for the spectra of model films prepared from HMO and neat HPSS, showing analogous peaks with only minimal deviations [39]. The HSQC spectrum also supports the assignments and the presence of polyether chains crosslinked with PSS. Specifically, the following correlations are evidenced: =CHR carbon signals at 128.07 and 125.39 ppm with hydrogen signals in the aromatic region; −CH_2_R carbon signals at 74.19, 70.38, 69.54, and 64.95 ppm with hydrogen signals of CH_2_ at 3.23 ppm (−CH_2_OH), 3.81 ppm (−CH_2_OSO_2_−), and 3.54–3.37 ppm, respectively; =CHR carbon signals at ca. 40 ppm with hydrogen signals in PSS aliphatic chains; and −CH_3_ carbon signals at 16.01 and 15.54 ppm with hydrogen signals at 0.738 ppm, assigned to lateral −CH_3_ groups in polyether chains. In the ^1^H NMR spectrum, the relative areas of the signals assigned to the aromatic rings are in excess in relation to those assigned to the crosslinking nodes (hydrogens in −CH_2_−S nodes). The relative integration areas are 12:12:2, corresponding to the aromatic CH protons (12:12) and CH_2_ in crosslinking nodes (−CH_2_−S−). This is consistent with HPSS chains, where one in six aromatic rings is crosslinked with polyether chains. Alternatively, “free” HPSS (HPSS containing non-crosslinked units) may also be contained in the supernatant liquid.

In the ^1^H spectrum of WS samples (Figure 2b), the nonappearance of peaks in the aromatic region indicates the absence of PSS in this sample. In agreement, the peak at 3.81 ppm, assigned to crosslinking nodes between the polyether and PSS, is also absent. Because the observed peaks resemble those attributed to polyether chains containing both hydroxymethyl (−CH_2_OH) and ether groups (−CH_2_OR) as substituents, the spectrum is consistent with polyethers similar to those found in the SL but lacking linkages with PSS. Hence, NMR analysis shows that HMO polymerizes within the PEDOT:PSS medium during the first annealing step at 50 °C for 24 h, yielding polyether chains composed of different blocks (statistical copolymers) grafted with PSS chains through −CH_2_OSO_2_- nodes. Thus, it can be concluded that HMO reacts similarly to when in spin-cast films subjected to 120 °C, as found in our previous studies. In addition, non-crosslinked polyether chains are also formed, and they are, at least in part, removed during the water-rinsing step. The presence of polyether chains unbounded to PSS can be explained by the little excess of HMO in relation to PSS in the mixtures (Table S1 in Supplementary Materials). It should be remarked that the reaction products derived from the HMO remaining within the PEDOT:PSS matrix (not extracted to the SL and WS) could not be characterized due to the insolubility of the films. However, in the regions attributed to the polyether chains, the ^1^H NMR spectra of the SL and SW samples are very similar to those obtained for soluble films prepared from pure HPSS and HMO in our previous work [39] and to the spectrum reported for the branched polymer from 3-hydroxymethyl-3-ethyl-oxetane, for which molecular weight analysis characterized the samples as branched polyethers with low to moderate molecular weights [46].

In order to further elucidate the film formation process, we compared the mass of the final dried films (after the water-rinsing process) with that of the starting materials, that is, HMO and PEDOT:PSS, considering that the aqueous dispersion of PEDOT:PSS has approximately 1.15 wt.% solids (according with the supplier). We found that the masses of the films prepared from the “0.005”, “0.015”, and “0.05” formulations are relatively close, between 27 and 31 mg, and represent, respectively, approximately 56%, 50%, and 47% of the mass of the starting materials used in their formulations. The increased mass loss with HMO content in the mixtures indicates that more polyether compounds have been removed in the films from the most concentrated formulations (in HMO) and suggests that the polyether/PEDOT:PSS ratios in the final films converge to similar values. This hypothesis is also in accordance with the final films’ thicknesses (Table 1) falling in a narrow range of values.

Hence, these results contribute to the interpretation of both the enhanced conductivities and structural sustainability obtained for the free-standing films. Before being subjected to water rinsing, the presence of PSS in the supernatant liquid indicates that the PSS content is reduced in the free-standing films compared to pristine PEDOT:PSS samples. Moreover, the remaining PSS within the films is likely crosslinked with polyether chains (POx-graft-PSS), similar to that existing in the supernatant liquid. Thus, the polymer products are expected to reduce the coulombic interactions between positively charged PEDOT chains and PSS, given that PSS is no longer negatively charged. Non-crosslinked polyether chains containing hydroxyl groups are also abundant within the films prior to water rinsing, as determined by the NMR analysis of WS samples. These chains tend to induce a screening effect between the negative PSS and the positively charged PEDOT by forming strong hydrogen bonds with the negative sulfonate groups in PSS. The weakening of the coulombic attraction between PEDOT^+^ and PSS^−^ facilitates the phase separation of the two polymers, where the segregation of excess PSS leads to PEDOT segregating in more densely packed and interconnected domains, facilitating charge transport. Therefore, both types of polymers, POx and POx-graft-PSS, should contribute to the observed conductivity enhancements. Other authors also attributed the conductivity improvement in PEDOT, when using PEG and EG (ethylene glycol) as additives, to the interaction between hydroxyl groups and negative PSS [47,48]. The conductivity’s decreasing trend with the increase in HMO content in the mixtures is likely due to an increased proportion of inherently insulating polyether compounds at the film surface, interrupting percolation paths between conducting PEDOT domains. Upon water rinsing, the removal of polyether compounds likely contributes to the further increase in conductivity by reducing such disruptions. Additionally, water-induced morphological alterations within the PEDOT:PSS network may also take place and contribute to enhancing the films’ conductivity. 

Regarding the effect on the structural stability of the films, the presence of polymer products formed from HMO in the films, either crosslinked or non-crosslinked with PSS, turns the PEDOT:PSS network into a more complex matrix composed of extra polymer compounds that interact with the original constituents PEDOT and PSS. The formed polyethers can solvate positive charges in PEDOT through oxygen in ether groups and interact with negative groups in PSS through the hydroxyl groups. Such effects can make them act as compatibilizers to the PEDOT:PSS network and stabilize the different phases formed within the PEDOT:PSS blend.

### 3.3. FTIR Spectroscopy Analysis

**Figure 3:**
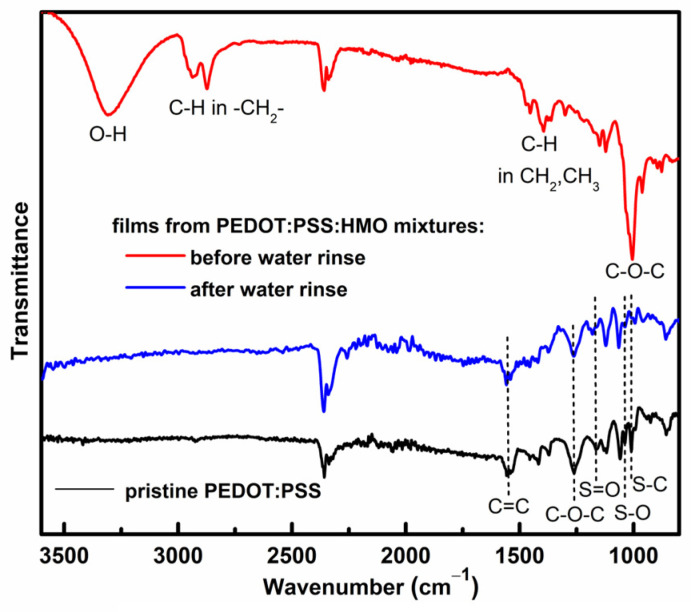
ATR-FTIR spectra of free-standing films prepared from HMO:PEDOT:PSS mixtures (0.050 *v*/*v*) before and after water rinsing and the spectrum of pristine PEDOT:PSS (powdered sample) after drying at 120 °C.

FTIR has often been used to investigate the role of additives and processing conditions on the chemical structure of PEDOT:PSS [30,37]. Hence, the films were examined in their dried forms (after drying at 120 °C) before and after being subjected to the water-rinsing step. Because the variances in the spectra with the HMO content mostly reflect the change in the mixtures’ compositions, the spectra of the films from the mixtures with the highest content of HMO (0.050 *v*/*v*) are taken as the most representative of the effect of the HMO additive (Figure 3 and Figure S5 in Supplementary Materials). As shown in Figure 3, the ATR-FTIR spectrum of the film not subjected to water rinsing is largely dominated by peaks assigned to polyether chains substituted with hydroxyl groups. 

The peaks at 3307, 2930–2870, 1445–1303, and 1008 cm^−1^ are attributed, respectively, to O−H stretching, C−H stretching in −CH_2_− groups, C−H bending in methylene and methyl groups, and C−O−C stretching vibrational modes. Interestingly, after water rinsing, the peaks assigned to polyether chains are not visible, and the spectrum resembles that of pristine PEDOT:PSS but with slight differences in the intensity of peaks assigned to PSS, namely, those attributed to S=O and S−O and S−C modes. The spectrum shows the peaks characteristic of PEDOT:PSS: 1600–1350 cm^−1^ (C=C stretching in thiophene and phenyl rings), 1260 cm^−1^ (C−O−C in ethylenedioxyl group), 1163 cm^−1^ (S=O in PSS), 1125 and 1060 cm^−1^ (C−O−C in in ethylenedioxyl group), 1040 cm^−1^ (S−O in PSS), 1008 cm^−1^ (S−C in PSS), and 858 cm^−1^ (C−S) [43,49]. The lower relative intensity of the peaks assigned to PSS, 1163, 1040, and 1008 cm^−1^, indicates that the PSS content is reduced in the free-standing films compared to the pristine PEDOT:PSS. This is in agreement with the NMR studies showing that the supernatant liquid sample (left behind in the film preparation process) contains PSS. Due to the relatively shallow penetration depth of ATR-FTIR (typically in the range of 0.5 to 1 μm), the spectra accurately represent only the surface of the films. Therefore, transmission FTIR spectroscopy was conducted to better investigate the entire film composition (Figure S5). In the spectra (Figure S5), the peaks attributed to hydroxyl-substituted polyether chains are prominent, although those assigned to PEDOT:PSS can be distinguished, as expected. This reveals that the polyether compounds are not fully removed during the water-rinsing step and that they remain mostly within the bulk of the films. Notably, upon water rinsing, the region attributed to C−O−C ether groups in polyether chains shows a narrower band, suggesting that a more homogeneous composition in terms of polyether compounds is left within the PEDOT:PSS network. In addition, a peak at 1383 cm^−1^, indicating the presence of an aromatic sulfonate ester, is revealed [50]. Hence, it can be concluded that the water-rinsing step primarily removes non-crosslinked polyethers, while the crosslinked chains (POx-graft-PSS) remain in the film, likely due to their lower hydrophilicity or the formation of a polymer network. These changes in the film spectra are consistent with the NMR spectra of the SL and WS samples, which show polyether compounds with and without crosslinking nodes with PSS.

### 3.4. Thermal Behavior

**Figure 4:**
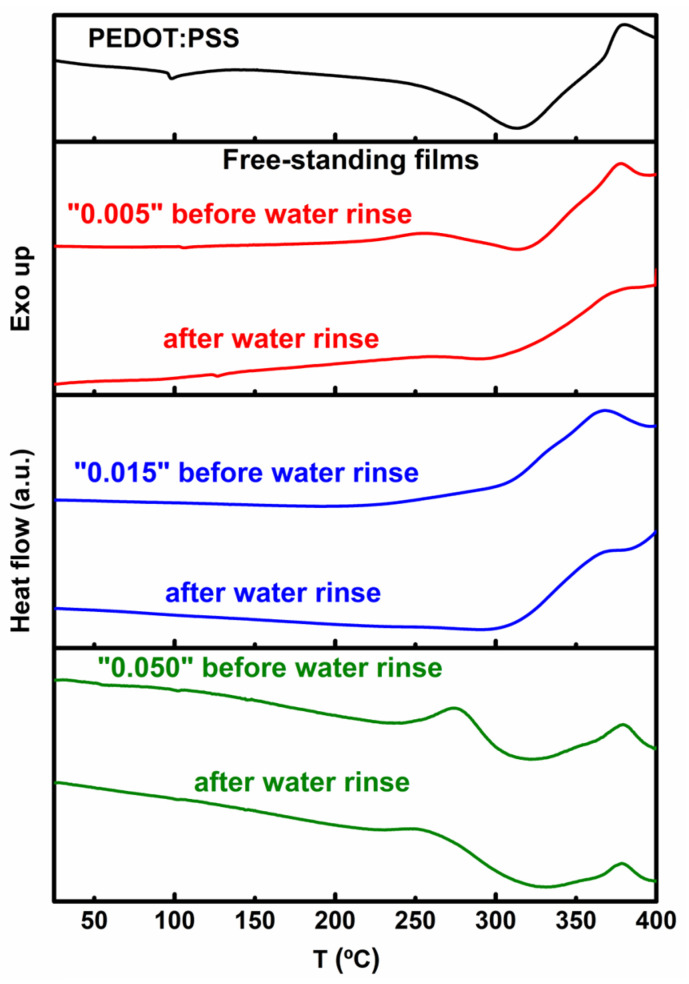
DSC thermograms of the free-standing films prepared from HMO:PEDOT:PSS mixtures with various HMO contents (HMO/PEDOT:PSSaq. = 0.005, 0.015, and 0.050, *v*/*v*) before and after water rinsing and compared with pristine PEDOT:PSS after drying at 120 °C.

DSC was employed to investigate the thermal properties of the produced free-standing films. Figure 4 shows the thermograms of the films before and after being subjected to water rinsing and upon drying at 120 °C. Only thermograms of the second heating are considered, since in the first heating (20–125 °C) there is a predominant peak of residual water loss, which disappears in subsequent runs [29].

In the thermogram of pristine PEDOT:PSS, three transitions are identified: a subtle step change in the DSC curve at about 92 °C, one broad endothermic signal peaking at 313 °C, and one exothermic signal at higher temperatures, peaking at 380 °C. The endothermic signal below 100 °C can be attributed to a glass transition, although glass transitions in PEDOT:PSS samples are often difficult to detect due to the complex nature of the interactions between the two polymers [29]. The absence of thermal events up to around 200 °C is justified by the quasi-amorphous state of pristine PEDOT:PSS [51,52]. Thermal transitions occurring over temperatures ranging between 300 °C and 400 °C have been attributed to melting accompanied by the thermal degradation of PEDOT [53] and PSS [54], respectively. In general, thermogravimetric analysis (TGA) has shown that PEDOT is stable up to 220 °C, and complete decomposition occurs around 390 °C [55]. By comparison, the thermograms of the free-standing films show noticeable differences. The peaks signaling glass transitions are less defined, which reflects changes in molecular structure and/or intermolecular organization. In the case of the free-standing films, crosslinking with PSS can restrict the mobility of the polymer chains and potentially increase the glass transition temperature or even suppress the glass transition. On the other hand, blending with the polyethers present in the samples can result in complex thermal behaviors, where both the glass transitions and other transitions become less apparent. For the films prepared from the mixtures with the highest HMO contents, 0.015 and 0.050, a broad and smooth endothermic transition can also be observed before the melting of PEDOT:PSS. This transition is more pronounced in films prepared from the highest concentration, peaking at about 228 °C. Therefore, it is likely associated mostly with polyether compounds, which are more abundant in these samples. The formed polyethers are expected to be less ordered than PEDOT:PSS due to the differences in their molecular structures and intermolecular interactions. Typically, polymer chains in polyethers, such as polyethylene glycol (PEG), are flexible, with few rigid segments, which leads to little orderly packing and low melting temperatures. This difference in thermal behavior impacts the processing and application of the polyether–PEDOT:PSS composite films. In particular, the increased polymer chain mobility at lower temperatures can be advantageous for applications that require greater flexibility, as, for instance, in flexible electronics or biomedical devices.

### 3.5. Surface Morphology Analysis

**Figure 5:**
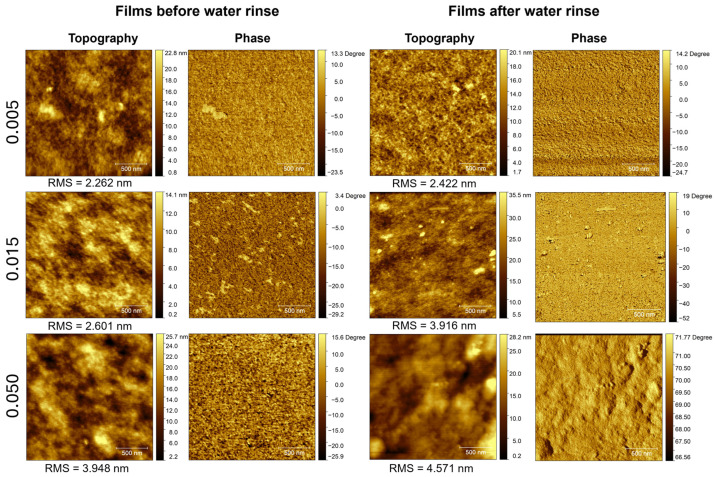
AFM images (2 μm × 2 μm) of free-standing films prepared from mixtures with different contents of HMO (HMO/PEDOT:PSSaq. = 0.005, 0.015, 0.050 *v*/*v*) before and after water rinsing. All samples were dried at 120 °C for 3 h prior to AFM characterization.

The surface morphology of the free-standing films was investigated by AFM. Figure 5 and Figure S6 in Supplementary Materials show topography and phase images obtained for the films before and after being subjected to water rinsing and after drying at 120 °C, with 2 μm × 2 μm and 5 μm × 5 μm scan areas, respectively.

The images show relatively rough surfaces, with the root-mean-square roughness (RMS) increasing both with the HMO content in the mixtures and after subjecting the films to water rinsing. Spin-cast films of pristine PEDOT:PSS PH1000 exhibit smoother surfaces, with RMS values less than 1.9 nm [41]. Only the films produced from the mixtures with the lowest content of HMO and subjected to water rinsing resemble the typical morphology of pristine PEDOT:PSS films, revealing a fibrillar-like interconnected structure, although their surface roughness is higher. Additionally, the films exhibit grainy surfaces, which are more evident in the larger-area images in Figure S6. A granular-type structured surface was also observed in HMO:PEDOT:PSS mixtures spin-cast over glass substrates in our previous studies [39]. The contrast observed in the phase images reveals different mechanical properties (such as viscoelasticity, adhesion, and friction) at the grains, indicating a different composition. This is more pronounced in non-rinsed films. Upon water rinsing, although the phase images indicate a more homogeneous composition, as the contrast is mostly due to topographical variations, the surface roughness increases, and grainy structures can still be distinguished in the topography images. Considering the ATR-FTIR spectra of the non-rinsed films and the NMR spectra of the washing solution, both of which identify polyether compounds, it can be inferred that the grains on the film surfaces are mainly composed of segregated polyether compounds and that these are dissolved and removed during the water-rinsing process. This agrees with the higher conductivity values found for the films after water rinsing since polyether compounds may interrupt percolation channels, as previously mentioned. Additionally, it is likely that the rinsing also affects the inner part of the film and removes polyethers within, which, upon subsequent domain adjustment, contributes to a rougher surface. Notably, the most conducting films of the series, i.e., those prepared from the mixtures with the highest HMO/PEDOT:PSS ratio subjected to water rinsing, appear to show the more pronounced phase separation between PEDOT and PSS, since more elevated regions on the PEDOT:PSS film surface are assigned to clusters enriched in conducting PEDOT, while surrounding lower-level (darker) areas are assigned to PSS-enriched insulating shells, in accordance with several studies. Thus, we propose that the higher polyether content in these films leads to more pronounced phase segregation between conducting PEDOT-enriched domains and insulating PSS-enriched domains, resulting in denser packing of PEDOT chains, thereby improving the charge transport.

### 3.6. Mechanical Properties

To evaluate the mechanical properties of the films, which are helpful in determining how materials might be used in electronics, tensile tests were performed on the films after they were rinsed and dried at 120 °C. The stress–strain curves indicate that the materials only suffered elastic deformation. The average values obtained for Young’s modulus, ultimate tensile strength, and elongation at break are presented in Table 2. In terms of stiffness, the increase in the concentration of HMO in the mixtures did not significantly affect the behavior of the films. However, for the two most concentrated formulations, the tensile strength and elongation at break of the films are enhanced, which may be due to a slightly higher content of POx-graft-PSS within the films. It is noteworthy that the dispersion of the results is superior for the films obtained from the mixtures with the highest concentration of HMO. This can be related to the higher variability in the thickness of these films (see Table 1). The values obtained for Young’s modulus are of the same order of magnitude as those found for films prepared from PEDOT:PSS and PEG-400 at a low concentration (1%) [56], although the ultimate tensile strength is inferior. However, the characteristics of the films did not compromise their handling or integrity during the measurement of the ECG signal, ensuring their suitability for the intended application.

To better elucidate the effect of HMO on the free-standing films’ performance as compared with other additives, Table 3 compiles the conductivity values and tensile properties of free-standing films, as reported in other works. For this comparison, studies where the free-standing films were deposited on polymer substrates (e.g., elastomers) were not considered (i.e., only works where the properties were measured in films not supported on any substrates were included).

**Table 3:** The most improved conductivity and tensile properties of free-standing films (average values) in this work and as reported in the literature. Nr: value not reported.

Additive	σ (Scm^−1^)	Young’s Modulus (MPa)	Ultimate Tensile Strength (MPa)	Elongation at Break (%)	Ref.
HMO	106	300	11	4	this work
PEG20K (50 wt.%)	101	Nr	~5	~25	[33]
PEO100K (44.4 wt.%)	74.7	Nr	~13	~20	[33]
PEO1000K (60.0 wt.%)	57.7	Nr	~7	36.8	[33]
PVA89K (66.7 wt.%)	0.14	Nr	~40	54.7	[33]
PEO100K (44.4 wt.%) +5 vol.% DMSO	238	Nr	Nr	~20	[33]
PEO100K (44.4 wt.%) +3 vol.% EG	245	Nr	Nr	~20	[33]
PVA89K (66.7 wt.%) +5 vol.% DMSO	142	Nr	Nr	51	[33]
PVA89K (66.7 wt.%) +3 vol.% EG	172	Nr	Nr	47	[33]
WPU (60 wt.%)	185	434.7	~7	11.6	[34]
PEG200 (4 vol.%)	1415.7	Nr	Nr	Nr	[35]
H_2_SO_4_	2500	Nr	Nr	Nr	[37]

From Table 3, it can be concluded that the improvement in conductivity caused by HMO is the same order of magnitude as or a little larger than those caused by the polyether compounds PEG20K (Mn = 20 K), PEO100K (Mn = 100 K), and PEO1000K (Mn = 1000 K) [33]. However, the elongation at break of the films produced with HMO is significantly lower. This may be related to differences in the chemical formula and molecular weight (not controlled in this study) of the polyethers formed from HMO when compared with those polymers. After the treatment with secondary dopants (DMSO or EG), the conductivity of the polymer blend films with such polymers or PVA was further increased, reaching a maximum value of 245 Scm^−1^ for PEO100K with 3 vol.% of EG, while keeping approximately the value for the elongation at break. Interestingly, PEG with lower molecular weight (PEG200) was reported to yield much greater enhancements in conductivity, reaching 1415.7 Scm^−1^, although the mechanical properties of the films were not reported [35]. Remarkably, the blending of PEDOT:PSS with waterborne polyurethane (WPU) can produce films with both good conductivity and tensile properties, although the elongation at break values are lower than those reported for PEG, PEO, and PVA. As observed, the maximum conductivity for free-standing films is achieved for films processed with H_2_SO_4_. In that work, the authors reported a multi-step process to produce free-standing films from mixtures of PEDOT:PSS and dilute H_2_SO_4_ in which the films are immersed in a concentrated H_2_SO_4_ solution for a week [37].

### 3.7. Application as Electrodes in ECG Biosensors

**Figure 6:**
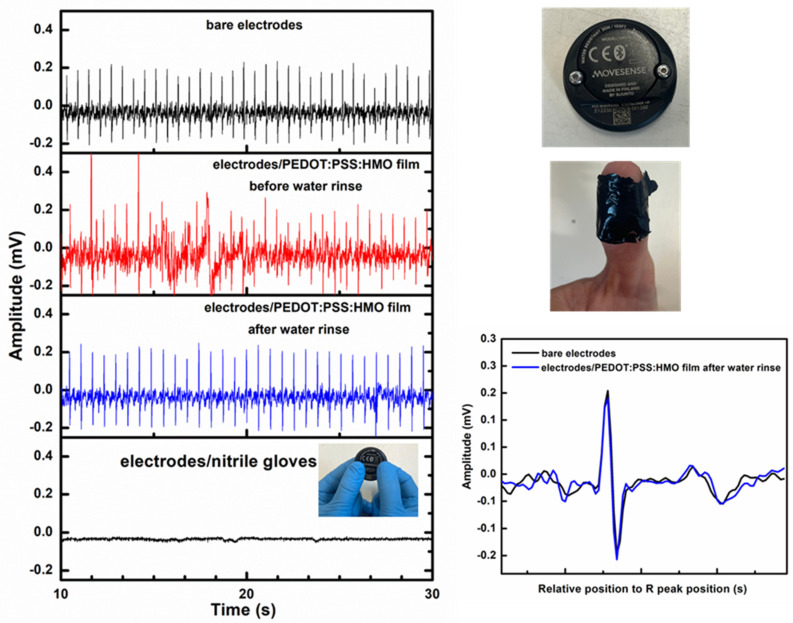
ECG signals obtained with a Movesense sensor device using films of PEDOT:PSS:HMO (HMO/PEDOT:PSSaq. = 0.050 *v*/*v*) interfacing with the human body (fingertips) and a comparison with bare electrodes and an electrically insulating interface (nitrile gloves) (**left**); photographs of the Movesense device used in this study and of a free-standing film covering the fingertip, as applied during the measurements (**top right**); ECG waveform obtained from PEDOT:PSS:HMO films in comparison with bare electrodes (**bottom right**).

In order to assess the viability of the films prepared from the PEDOT:PSS:HMO mixtures as dry, flexible electrodes for wearable electronics, they were used in ECG signal detection essays. Electrocardiography (ECG) is an important technique for analyzing and monitoring cardiovascular physiological conditions (e.g., arrhythmia). Thus, films prepared from the “0.050” PEDOT:PSS:HMO mixtures, before and after being subjected to the water-rinsing process and in their dried form, were tested as the interface between the human body (the fingertip) and the electrodes of a commercial ECG 1-channel biosensor device. For this, a commercial Movesensor Medical Sensor was chosen because it allows the data analysis as a single-channel ECG and comes with an app for real-time measuring and viewing modes. Importantly, the films can adapt to the naturally curved fingertips, promoting a high response in electronic signals. For comparison, the tests were repeated using the biosensor device interfacing directly with the human body, specifically with the left and right thumbs touching its bare electrodes, and with the user wearing nitrile gloves, taken as the insulating control sample (Figure 6).

As observed, in the case of the films subjected to water rinsing, the measurements closely track the reference test in which the electrodes pertaining to the biosensor interface directly with the fingertips (bare electrodes). As expected, with an insulating interface (nitrile gloves), no EGC signal is recorded. Nevertheless, a less regular plot is found when the films that were not subjected to the water-rinsing process are used. This should be due to the presence of excess insulating polyethers at the film surface, as indicated by the ATR-FTIR spectra. Thus, the tests show that the water-rinsing process is crucial for improving the films´ performance as electrodes. Future work will be carried out with other exercise modalities and several users to better assess the films’ performance as electrodes in EGC recording. Still, these results indicate that the developed films hold promise for applications in health monitoring.

## 4. Conclusions

In summary, a significant increase in electrical conductivity of approximately two orders of magnitude and enhanced structural stability enabling free-standing films have been demonstrated for thick films cast from mixtures of PEDOT:PSS and an oxetane additive (HMO). NMR and FTIR studies revealed that HMO polymerizes within the PEDOT:PSS medium to produce polyether compounds, including graft copolymers with covalently bonded PSS. The enhanced conductivity is attributed to the phase separation between conducting PEDOT chains and PSS, resulting from weakened coulombic forces between PEDOT and PSS due to the crosslinking and hydrogen bonds formed between polyether chains and PSS. The segregation of excess PSS is likely to promote more densely packed and interconnected PEDOT-enriched domains, thereby facilitating charge transport. Rinsing the films in water removes non-crosslinked polyether compounds (without PSS), reducing the film thickness and exposing more PEDOT at the film surface, further increasing the conductivity. Ultimately, the most conductive films, displaying conductivity around 106 Scm^−1^, show potential for applications as dry and flexible electrodes for ECG monitoring.

## Figures and Tables

**Table 1:** Conductivity and thickness of free-standing films prepared from HMO:PEDOT:PSS mixtures measured before and after the water-rinsing step.

HMO/PEDOT:PSSaq.(*v*/*v*)	Conductivity(Scm^−1^)		Thickness(μm)	
	Before rinse	After rinse	Before rinse	After rinse
0.005	49 ± 6	73 ± 10	45 ± 17	33 ± 5
0.015	32 ± 3	72 ± 8	42 ± 4	40 ± 9
0.050	8 ± 1	106 ± 15	202 ± 11	55 ± 10
0 (pristine PEDOT:PSS)	1.505 ± 0.003	-	3.3 ± 0.5	-

**Table 2:** Tensile properties of the free-standing films prepared from HMO:PEDOT:PSS mixtures measured after the water-rinsing step and upon drying at 120 °C.

HMO/PEDOT:PSSaq (*v*/*v*)	Young’s Modulus (GPa)	Ultimate Tensile Strength (MPa)	Elongation at Break (%)
0.005	0.4 ± 0.1	7.8 ± 0.9	2.3 ± 0.8
0.015	0.5 ± 0.1	12 ± 3	3 ± 1
0.050	0.3 ± 0.3	11 ± 7	4 ± 2

## Data Availability

The raw data supporting the conclusions of this article will be made available by the authors upon request.

## Supplementary Materials

The following supporting information can be downloaded at https://www.mdpi.com/article/10.3390/polym16162292/s1: Figure S1: Photographs of the developed films during the annealing step at 50 °C showing the supernatant liquid (left photo) and after the second drying treatment at 120 °C (right); Figure S2: ^13^C APT NMR spectra of SL in D_2_O; Figure S3: ^13^C APT NMR spectra of WS sample in D_2_O; Figure S4: HSQC spectrum of WS in D_2_O; Table S1: The stoichiometry of PH1000 PEDOT:PSS in the prepared mixtures; Figure S5: Transmission FTIR spectra of the free-standing films prepared from HMO:PEDOT:PSS mixtures (0.050 *v*/*v*) before and after water rinsing upon drying at 120 °C; Figure S6: AFM images (5 μm × 5 μm) of free-standing films prepared from mixtures with different contents of HMO (HMO/PEDOT:PSSaq. = 0.005, 0.015, 0.050 *v*/*v*) before and after water rinsing. All samples were dried at 120 °C for 3 h.
