# Supplementary material for: Free-Standing, Water-Resistant, and Conductivity-Enhanced PEDOT:PSS Films from In Situ Polymerization of 3-Hydroxymethyl-3-Methyl-Oxetane
Source: Polymers (Basel). 2024 Aug 14;16(16):2292. doi: 10.3390/polym16162292 (PMC11360818; doi:10.3390/polym16162292)
Supplement: Supplementary file 1 [file polymers-16-02292-s001.zip › polymers-3081615-supplementary.pdf]

Supplementary Materials

Free-standing, water-resistant, and conductivity-enhanced PEDOT:PSS films from in-situ polymerization of 3-hydroxymethyl-3-methyl-oxetane

Sara M. Jorge [1,2], Luís Santos [2], Maria João Ferreira [2], Carolina Marto-Costa [2], Ana Paula Serro [2,3], Adelino M. Galvão [2], Jorge Morgado [1,4] and Ana Charas [1]*

[1] Instituto de Telecomunicações, Instituto Superior Técnico, Av. Rovisco Pais, P-1049-001, Lisboa, Portugal

[2] Centro de Química Estrutural, Institute of Molecular Sciences and Departamento de Engenharia Química, Instituto Superior Técnico, Universidade de Lisboa, Portugal

[3] Egas Moniz Center for Interdisciplinary Research (CiiEM), Egas Moniz School of Health & Science, Campus Universitário, Quinta da Granja, 2829-511 Monte da Caparica, Almada, Portugal.

[4] Department of Bioengineering, Instituto Superior Técnico, Universidade de Lisboa, Lisboa, Portugal

* Correspondence: ana.charas@lx.it.pt (A. Charas)

Table S1. Stoichiometry in the prepared mixtures of PH1000 PEDOT:PSS.

HMO/PEDOT:PSSaq (v/v)	HMO[a] (mmol)	HMO/PEDOT:PSS[b] (mmol/g)	HMO/PEDOT[c] (mmol/g)	HMO/PEDOT[d] (mmol/mmol)	HMO/PSS[e] (mmol/g)	HMO/PSS[f] (mmol/mmol)
0.005	0.048	4.17	14.60	2.05	5.84	1.08
0.015	0.144	12.52	43.81	6.14	17.52	3.23
0.050	0.480	41.72	146.02	20.46	58.41	10.76

[a] to 1 mL of PEDOT:PSS aqueous dispersion (HMO purity is 98% and M.M. (HMO) = 102.13 gmol^{-1})
[b] mmol of HMO per gram of PEDOT:PSS polymers assuming solids content in PEDOT:PSS aqueous dispersion is 1.15 wt% (average of 1.0-1.3 wt%)
[c] mmol of HMO per gram of PEDOT polymer assuming PEDOT:PSS ratio = 1:2.5
[d] mmol of HMO per mmol of PEDOT repeat unit with M.M. (PEDOT) = 140.15 g.mol^{-1}
[e] mmol of HMO per gram of PSS polymer assuming PEDOT:PSS ratio = 1:2.5
[f] mmol of HMO per mmol of PSS repeat unit with M.M. (PSS) = 184.20 g.mol^{-1}

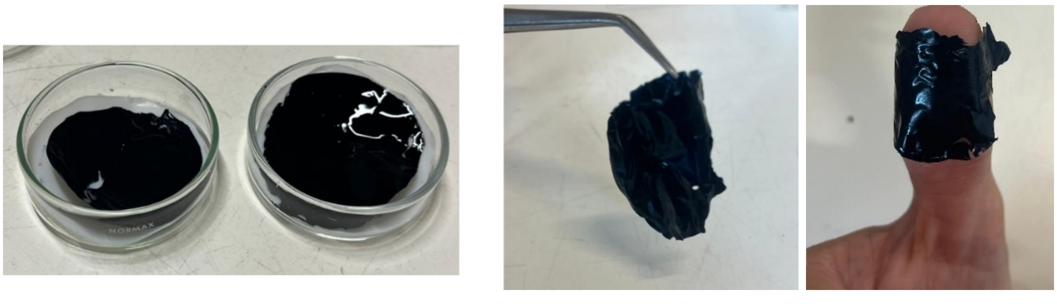

Figure S1. Photographs of the developed films during the annealing step at 50 °C showing the supernatant liquid (left photo) and after the second drying treatment, at 120 °C (center and right).

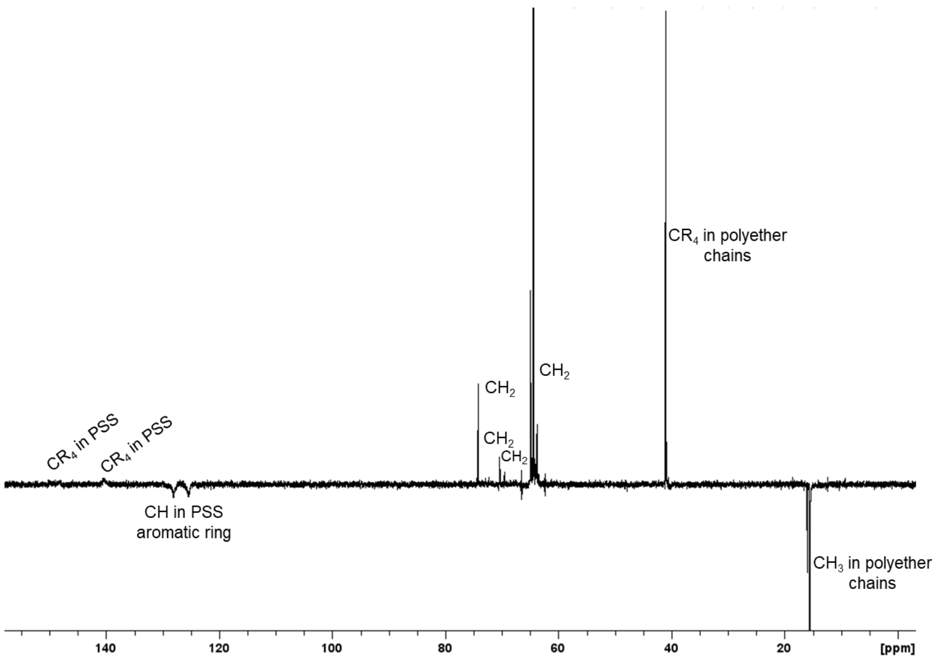

Figure S2. ^{13}C APT NMR spectra of SL in D_2O.

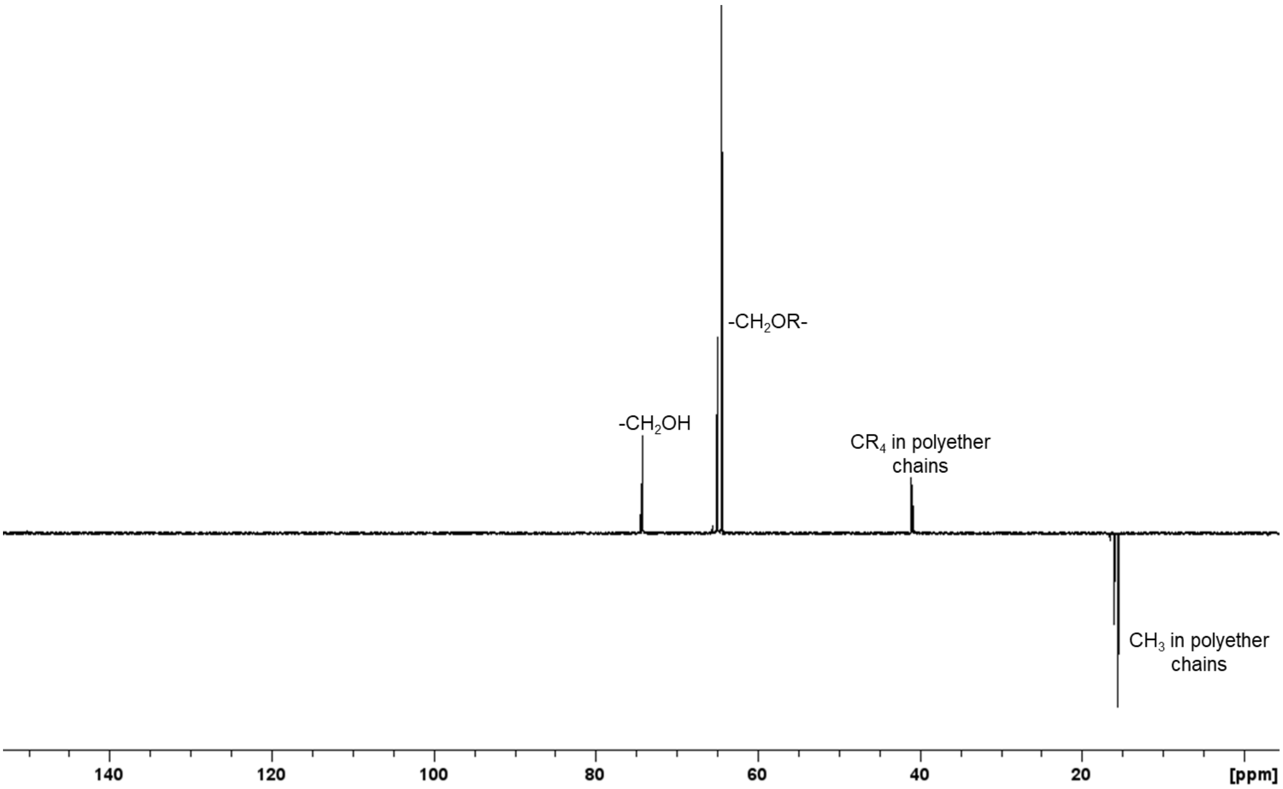

Figure S3. ^{13}C APT NMR spectra of WS sample in D_2O.

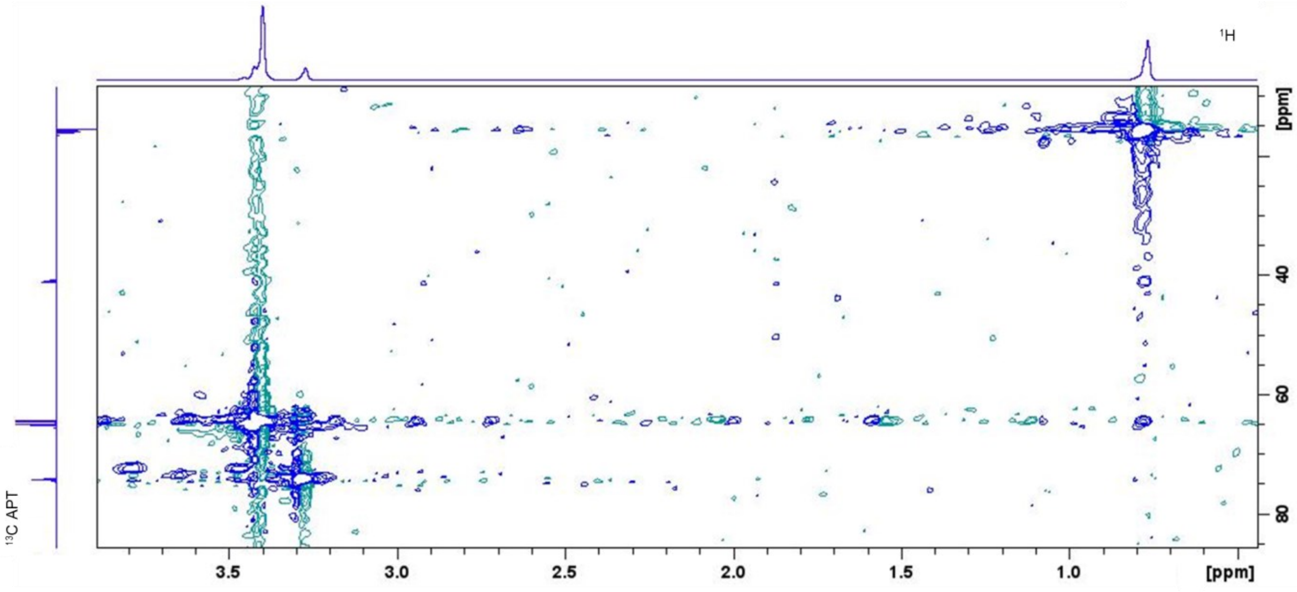

Figure S4. HSQC spectrum of WS in D_2O.

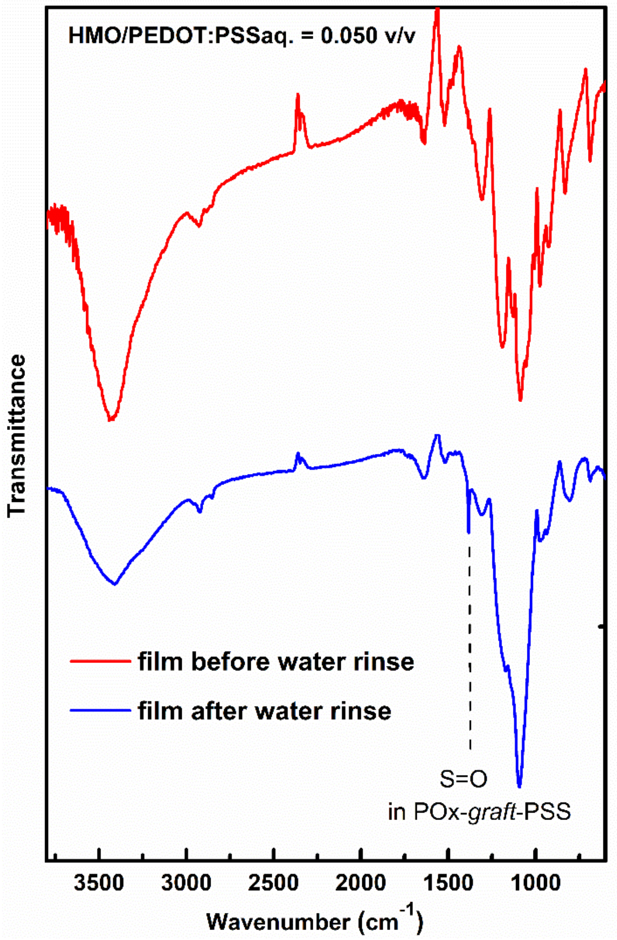

Figure S5. Transmission FTIR spectra of the free-standing films prepared from HMO:PEDOT:PSS mixtures (0.050 v/v) before and after water rinsing, upon drying at 120 °C.

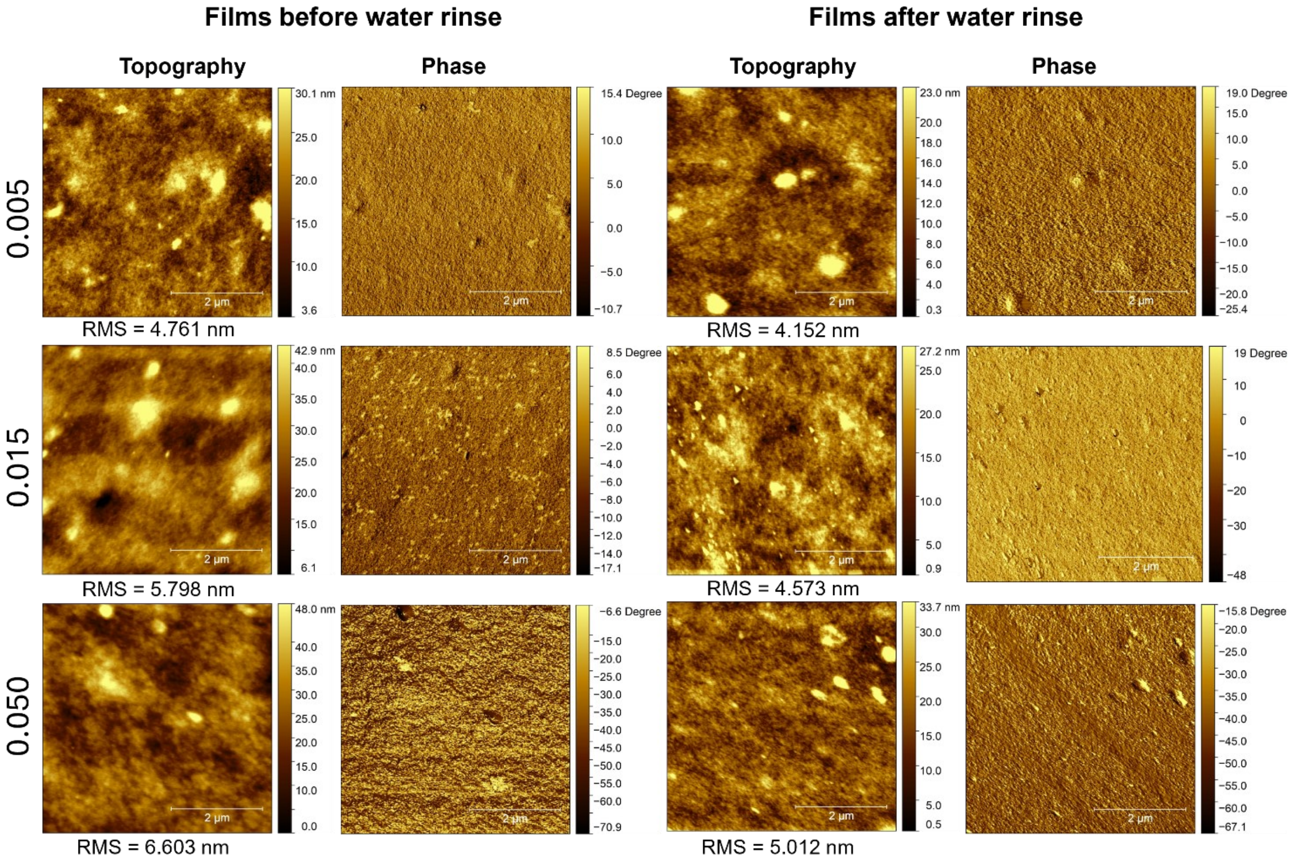

Figure S6. AFM images (5 µm × 5 µm) of free-standing films prepared from mixtures with several contents of HMO (HMO/PEDOT:PSSaq. = 0.005, 0.015, 0.050 v/v), before and after water-rinsing. All the samples were dried at 120 °C for 3 hours.
